# The easternmost discovery of the Mediterranean weevil *Pachyrhinuslethierryi* (Coleoptera, Curculionidae, Entiminae): Is a further invasion possible?

**DOI:** 10.3897/zookeys.799.29934

**Published:** 2018-11-28

**Authors:** Yakov N. Kovalenko, Evgeniy N. Akulov, Nikolai Yunakov

**Affiliations:** 1 University of Oslo, Natural History Museum, Department of Zoology, P.O. Box 1172, Blindern, NO-0318 Oslo, Norway A.N. Severtsov Institute of Ecology and Evolution Moscow Russia; 2 A.N. Severtsov Institute of Ecology and Evolution, Russian Academy of Sciences, 33 Leninskiy prosp., 119071, Moscow, Russia All-Russian Plant Quarantine Center Krasnoyarsk Russia; 3 All-Russian Plant Quarantine Center, Krasnoyarsk branch, 31A Maerchak str., 660075, Krasnoyarsk, Russia University of Oslo Oslo Norway

**Keywords:** Asia Minor, Crimea, invasive species, new record, Polydrusini, weevils

## Abstract

*Pachyrhinuslethierryi* (Desbrochers des Loges, 1875) is a Mediterranean weevil species that has become remarkably well known as a result of a series of recent introductions across Western and Central Europe. This species has recently reached Asia Minor and the Crimean Peninsula, as confirmed by several new records. The vectors of invasion in Crimea and possible further expansion are suggested.

## Introduction

The native range of *Pachyrhinuslethierryi* is along the Mediterranean coast of France, Corsica, Sardinia, and Sicily (Hoffmann 1950). Since the 1980s, *P.lethierryi* has spread rapidly as an adventive species into many European countries (Germann et al. 2005, 2013; Scholze 2007; Heijerman 2008; Delbol 2009; Barclay and Morris 2011, Yunakov 2013; Çerçi 2016; Germann and Braunert 2018).

## Material and methods

For spatial analysis we used occurrence datasets from the Global Biodiversity Information Facility (GBIF 2018) and the Ukrainian Biodiversity Information Network (UkrBIN 2018). The beetle and genitalia were photographed with a Zeiss SteREO Discovery.V20 microscope equipped with Canon EOS 5D Mark III camera. Spatio-temporal data have been mapped in QGIS v. 3.2 using a Google Maps satellite imagery layer. Male genitalia were placed in a transparent polypropylene tube with glycerine and pinned to the underside of the card with the mounted specimen.

### Collections

**RPQC** All-Russian Plant Quarantine Center, Moscow

**ZMUN** Zoological Museum University of Oslo, Norway

## Results and discussion

### Pachyrhinus (Pachyrhinus) lethierryilethierryi

Taxon classificationAnimaliaColeopteraCurculionidae

(Desbrochers des Loges, 1875)

Figures 1–3

#### Material examined.

1 f, Crimea, Yalta, Mt Dorsan, 44.5030N; 34.1601E, beating from *Thujaoccidentalis*, N. Yunakov leg., 01.ix.2013 (ZMUN); 1 m, Crimea, Sevastopol, Uchkuyevka, 44.6408N; 33.5367E, E.N. Akulov leg., 1–7.vi.2017 (RPQC).

Recent records indicate that *P.lethierryi* is continuing its expansion to eastward. The northernmost record to date is from Magdeburg, Germany, in 2013. In 2018, it was first recorded in Asia Minor at Urla, İzmir, Turkey (UkrBIN 2018). All specimens were found in urban areas with numerous *Cupressus*, *Thuja*, and *Juniperus* trees in neighbouring properties. These plants are known as the principal hosts for *P.lethierryi* (Hoffmann 1950; Alziar 1977; Germann et al. 2005; Plant et al. 2006) and are considered to be the main vectors for the further spread of *P.lethierryi* (Heijerman 2008). Recent records from Crimea are obviously in line with a general trend of this species’ dispersal northward and eastward with commercial ornamental plants. However, we have no proof that *P.lethierryi* has established a viable population in Crimea. Thus, it may be preliminarily characterized as a “Robinson Crusoe species”, that is, one that was accidentally introduced without further naturalization. A remarkable feature of adventive populations of *P.lethierryi*, along with some invasive *Otiorhynchus* species, is that no specimens are known from natural habitats.

In Asia Minor and Crimea, *P.lethierryi* can be easily confused with some species of the genera *Dichorrhinus* Desbrochers des Loges, 1875 and *Rhinoscythropus* Desbrochers des Loges, 1895. The following key is given to distinguish *P.lethierryi* from similar species:

**Figures 1–3.**
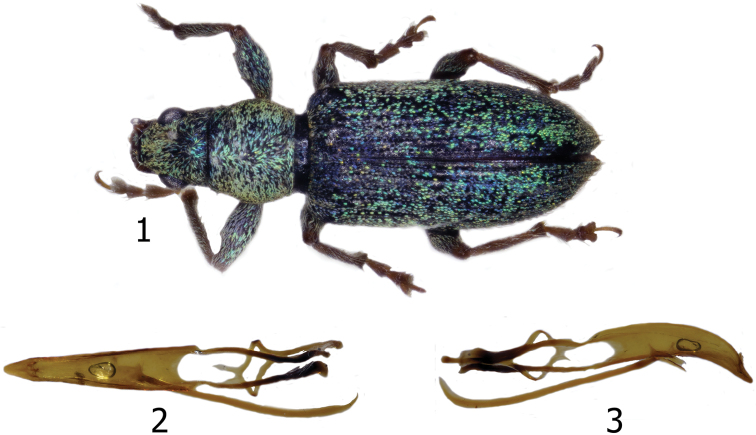
*Pachyrhinuslethierryi*. **1** Male, dorsal habitus **2** Aedeagus, dorsal view **3** Aedeagus, lateral view.

**Figure 4.**
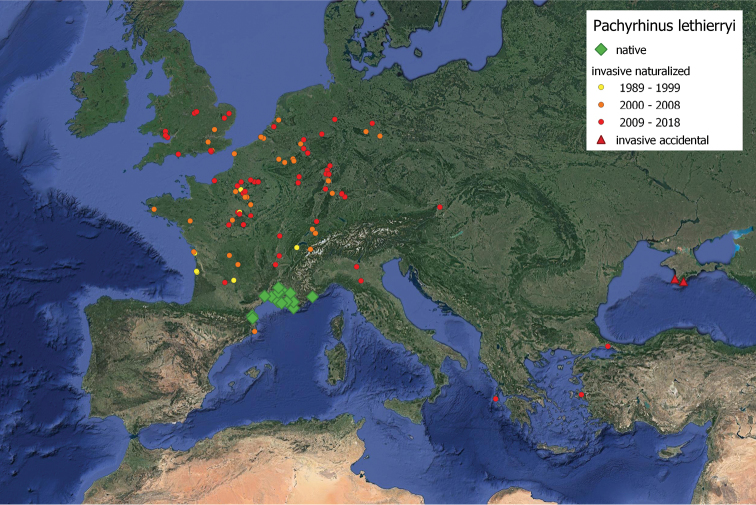
Occurrence pattern of *Pachyrhinuslethierryi*. The yellow through orange to red gradient indicates the chronology of invasion from 1989 to present.

**Table:** 

1	Antennal scrobes laterally open. Epifrons (rostral dorsum) between antennal base as wide as distance between eyes. Antennal scape slightly curved. Median lobe regularly narrowed apically, without subapical lateral callosities	**2**
–	Antennal scrobes dorsally open. Epifrons (rostral dorsum) between antennal base as wide as ½ distance between eyes. Antennal scape strongly curved. Median lobe constricted before apex, with subapical lateral callosities	**3**
2	Scales ovate, uniformly green. Frons (nasal plate) squamulate. Body with black erect or suberect pilosity. Body length to 5 mm	***Pachyrhinuslethierryi* (Desbrochers des Loges, 1875)**
–	Scales piliform, cupreous, forming spotty pattern on elytra. Body without black erect or suberect pilosity. Body length greater than 6 mm	***Pachyrhinussquamulosus* (Herbst, 1795)**
3	Frons (nasal plate) bare. Longitudinal diameter of eye equals 0.5–0.6 times distance between eyes	***Dichorrhinus* Desbrochers des Loges, 1875**
–	Frons (nasal plate) squamulate. Longitudinal diameter of eye equal to distance between eyes	***Rhinoscythropusvespertilio* (Faust, 1884)**

## Supplementary Material

XML Treatment for Pachyrhinus (Pachyrhinus) lethierryilethierryi
